# An Improved Nonlinear Capacitance Model for GaN HEMTs Based on the Angelov Model

**DOI:** 10.3390/mi16121318

**Published:** 2025-11-25

**Authors:** Yuchen Miao, Qingyu Yuan, Chuangye Wang, Jiali Cheng

**Affiliations:** 1 School of Electronic Engineering, Jiangsu Ocean University, Lianyungang 222005, China; 2023210615@jou.edu.cn (Y.M.); 2024210612@jou.edu.cn (C.W.); 2 Zhejiang Key Laboratory of Information and Energy Integrated Microsystem, Yangtze Delta Region Institute (Huzhou), University of Electronic Science and Technology of China, Huzhou 313001, China; 3 Zhejiang Provincial Key Laboratory of Spatial Information Perception and Transmission, Institute of Spatial Information, Hangzhou Dianzi University, Hangzhou 310018, China; qyyuan@hdu.edu.cn

**Keywords:** intrinsic capacitance, parameter extraction, nonlinear capacitance, small-signal equivalent circuit, Angelov mod

## Abstract

To accurately model the nonlinear characteristics of the intrinsic capacitance of Gallium Nitride High Electron Mobility Transistors (GaN HEMTs), this paper proposes an improved nonlinear capacitance model based on the traditional Angelov model. The experiments employed GaN HEMTs fabricated by United Monolithic Semiconductors (UMS) using the GH15-10 process. These devices feature a uniform gate length of 150 nm, with gate widths designed as 2 × 10 μm, 4 × 20 μm, 6 × 30 μm, and 8 × 40 μm, respectively (gate width is calculated as “number of gate fingers × unit gate width”). In this study, two types of devices with gate widths of 2 × 10 μm and 4 × 20 μm were selected to extract intrinsic capacitance parameters, which were then substituted into the traditional Angelov model and the proposed improved model, respectively. By comparing the coefficient of determination (R^2^) and Root Mean Square Error (RMSE) of the two models, it is found that the improved model has a significantly higher degree of agreement with the experimental measurement dates and is more suitable for the quantitative characterization of the nonlinear capacitance characteristics of GaN HEMTs. This research provides support for the accurate application of device models in the design of high-frequency circuits in subsequent studies.

## 1. Introduction

Two-dimensional (2D) materials, leveraging their atomically thin structure, ultra-high specific surface area, and excellent electronic transport properties, have broken through the bottlenecks of traditional semiconductors and provided a new pathway for the miniaturization and high-efficiency innovation of semiconductor devices [1]. Among them, GaN HEMTs based on 2D material modification or integration have been widely applied in power electronics and radio frequency (RF) fields due to their outstanding performance [2]. These devices can provide high output power, achieve high breakdown voltage, and exhibit excellent RF and microwave characteristics. An accurate large-signal model of GaN HEMTs is crucial for improving the accuracy and convergence of circuit simulation.

The key feature of 2DEG is that electrons are strongly confined by quantum mechanical effects in the dimension perpendicular to the interface (typically within a range of only a few nanometers), thereby forming an extremely thin (on the order of ~10 nm), high-concentration, and high-mobility electron gas layer. This two-dimensional confinement property significantly mitigates lattice scattering and interface roughness scattering, endowing electrons with extremely high low-field mobility. This operating mechanism is fundamentally different from that of MOSFETs, where an inversion layer is formed by inducing charges on the semiconductor surface via gate voltage. Therefore, targeted modifications to traditional large-signal empirical models are necessary to accurately describe the nonlinear capacitive behavior of GaN HEMTs.

Currently, research on the large-signal models of GaN HEMTs primarily focuses on the domain of DC models. In 2018, Jie Yang et al. proposed a non-square law model through an investigation of classical models originally developed for GaAs MESFETs and HEMTs, which notably improved the accuracy of DC I-V models for GaN HEMTs [3]. A lookup table-based GaN HEMT I-V model was constructed in 2021 [4]. By introducing a scalable scaling factor, this model substantially enhanced the accuracy and applicability of the DC I-V model. While the research on AC operating characteristics—namely Capacitance–Voltage (C-V) models (or Charge–Voltage models)—is relatively insufficient, in the design of RF/microwave high-frequency circuits, AC characteristics directly affect the switching speed, high-frequency response, and circuit stability of devices. Thus, establishing an accurate AC C-V model is particularly crucial. The current mainstream capacitor models are shown in the following Table 1.

From the above table, it can be observed that although physics-based models feature clear physical meanings, high accuracy, and a wide application range, they suffer from high modeling complexity and difficult parameter extraction, making it challenging to meet the requirements of fast simulation and iteration in high-frequency circuit design. However, empirical models have relatively narrow application scopes and insufficient physical interpretability. Therefore, building upon the conventional empirical Angelov model, this paper incorporates physical effects and proposes a nonlinear capacitance model for GaN HEMTs through the introduction of exponential functions and cross-coupling terms. The selected exponential function in this paper is not designed to fit the non-monotonic curve; instead, it is because $C_{gs}$ exhibits non-monotonic characteristics with $V_{gs}$, and the S-shaped feature of the exponential function can precisely reproduce this physical effect. Similarly, the cross-coupling term is not added merely to increase parameters, but to describe the coupling effect between $V_{gs}$ and $V_{ds}$ generated through the channel electric field and polarization charges.

## 2. Parameter Extraction

### 2.1. Extraction of Parasitic Capacitance

**Figure 1:**
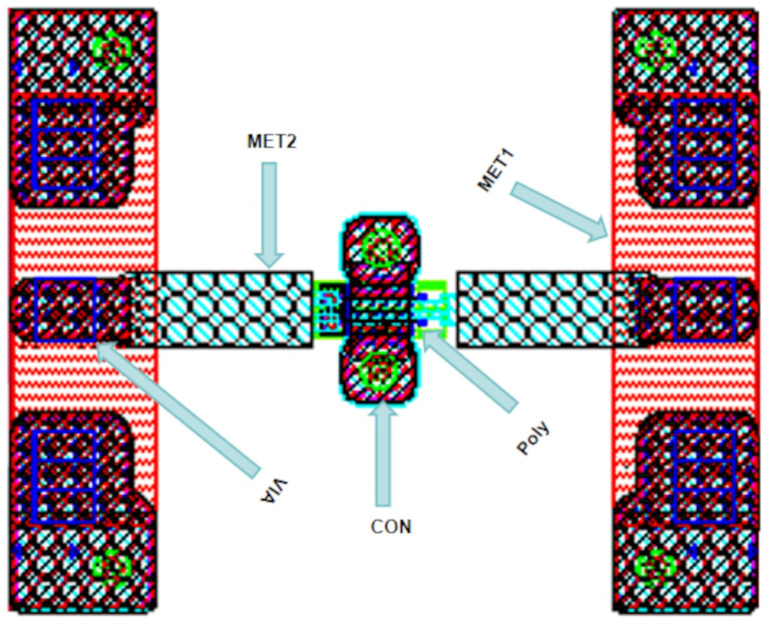
HEMT device test layout.

Figure 1 presents the HEMT device test layout. This paper adopts a method combining direct extraction and numerical optimization to extract model parameters [9,10]. Its core idea follows the workflow: extract parasitic capacitances de-embed parasitic capacitances extract parasitic inductances and parasitic resistances de-embed all parasitic parameters extract intrinsic parameters.

**Figure 2:**
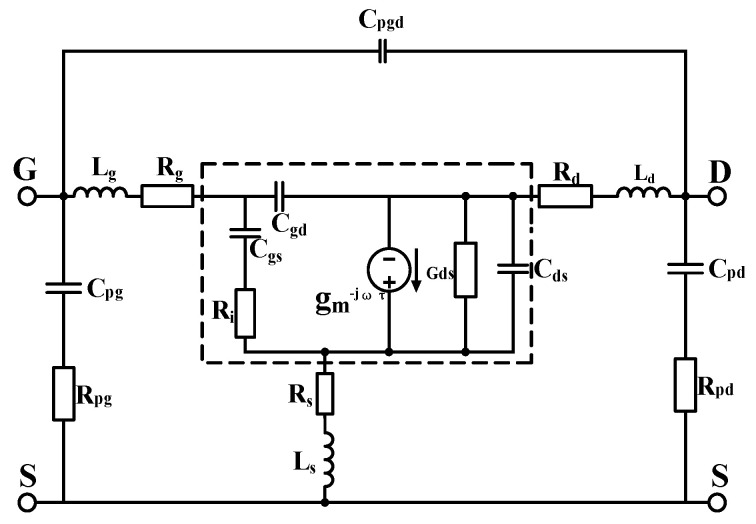
16-Element Small-Signal Equivalent Circuit Model.

With the continuous increase in the operating frequency of devices, especially when entering the millimeter-wave band, the impact of parasitic effects (such as substrate effects) on device characteristics can no longer be ignored. In this context, traditional small-signal models, which are mainly suitable for frequencies below 40 GHz, can hardly meet the requirements of current circuit design. Therefore, this paper uses a 16-element small-signal equivalent circuit model. Based on the traditional small-signal circuit model, parasitic components $R_{pg}$ and $R_{pd}$ are introduced to effectively characterize the substrate effect. The small-signal model is shown in Figure 2 below:

First, when a GaN HEMT device operates at low frequency (f < 5GHZ) and in a “cold” pinch-off state, the influence of parasitic inductance and parasitic resistance on the circuit can be ignored, and capacitance plays a major role. The topological structure of the small-signal equivalent circuit under this condition is shown in Figure 2. This paper adopts a parasitic capacitance extraction method based on the size scaling model. Compared with traditional extraction methods, it has no restrictions or assumptions on the equivalent circuit. By using four devices that conform to the size scaling relationship, all parasitic capacitances $C_{pg}$, $C_{pd}$ and $C_{pgd}$ can be extracted simultaneously without complex calculation and derivation processes.

The steps of the method based on the size scaling model are as follows: First, the small-signal S-parameters of devices with different gate widths are measured under the zero-bias condition. Second, the S-parameters are converted into Y-parameters. Finally, the values of parasitic capacitances are derived from the imaginary parts of the Y-parameters (admittance parameters).

The admittance matrix of Y-parameters is expressed as:

(1) $$Y=\left[\begin{array}{cc} \frac{jwC_{pg}}{1+jwC_{pg}R_{pg}}+jwC_{pgd} & -jwC_{pgd}\\ -jwC_{pgd} & \frac{jwC_{pgd}}{1+jwC_{pg}R_{pd}}+jwC_{pgd} \end{array}\right]$$

The method based on the size scaling model is as follows:

(2) $$C_{pg}={\left.\frac{Im\left(Y_{11}\right)}{w}\right|}_{w\to 0}-C_{pgd}$$

(3) $$C_{pd}={\left.\frac{Im\left(Y_{22}\right)}{w}\right|}_{w\to 0}-C_{pgd}$$

(4) $$C_{pgd}={\left.\frac{Im\left(Y_{12}\right)}{w}\right|}_{w\to 0}$$

In the formula, w represents the device’s gate width.

**Figure 3:**
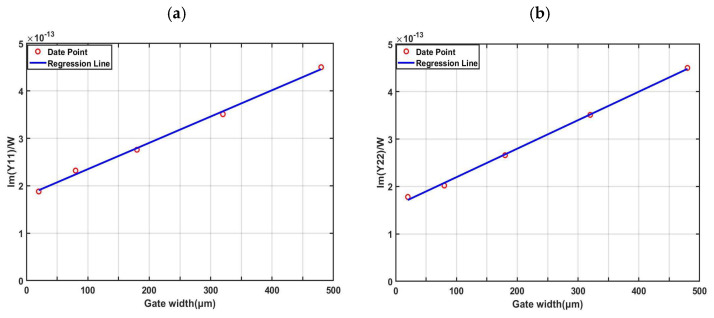
Extraction Results Based on the Size Scaling Model Method: (**a**) $C_{pg}+C_{pgd}$; (**b**) $C_{pd}+C_{pg}$.

By measuring the S-parameters of four different devices under the zero-bias condition [11,12], the average value within the low-frequency range (0.5–10 GHz) was taken as the final value. The relationship between parasitic capacitances and gate width is shown in Figure 3. Since the value of $C_{pgd}$ is extremely small and almost negligible, we can consider the intercept values as the values of $C_{pd}$ and $C_{pg}$ [13].

The value of the substrate resistance can be extracted and calculated using the following formula:

(5) $$R_{pg}=Re\left(\frac{1}{Y_{11}+Y_{12}}\right)$$

(6) $$R_{pd}=Re\left(\frac{1}{Y_{22}+Y_{12}}\right)$$

### 2.2. Extraction Method for Parasitic Inductance and Parasitic Resistance

This paper adopts the short-circuit test method [14], and the values of parasitic inductance and parasitic resistance can be obtained using the following formulas.

1. Convert the measured S-parameter matrix of the open-circuit structure and the S-parameter matrix of the short-circuit structure into the corresponding Y-parameter matrix $Y^{PAD}$ and $Y^{short}$.

2. Eliminate the influences of parasitic capacitance and substrate resistance using circuit analysis theory to obtain the Y-parameter matrix $Y^{short}$ that only contains the parasitic inductance and parasitic resistance of the lead.

(7) $$Y^{short1}=Y^{short}-Y^{PAD}$$

3. Convert the Y-parameter matrix $Y^{short1}$ to the Z-parameter matrix $Z^{short1}$:

(8) $$Z^{short1}=\left[\begin{array}{cc} R_{g}+jwL_{g}+R_{s}+jwL_{s} & R_{s}+jwL_{s}\\ R_{s}+jwL_{s} & R_{d}+jwL_{d}+R_{s}+jwL_{s} \end{array}\right]$$

4. Calculate the parasitic inductance and parasitic resistance values of the three leads using the following formulas.

(9) $$R_{g}=Re\left(Z_{11}^{short1}-Z_{12}^{short1}\right)$$

(10) $$R_{d}=Re\left(Z_{22}^{short1}-Z_{12}^{short1}\right)$$

(11) $$R_{s}=Re\left(Z_{12}^{short1}\right)$$

(12) $$L_{g}=\frac{Im\left(Z_{11}^{short1}-Z_{12}^{short1}\right)}{w}$$

(13) $$L_{d}=\frac{Im\left(Z_{22}^{short1}-Z_{12}^{short1}\right)}{w}$$

(14) $$L_{s}=\frac{Im\left(Z_{12}^{short1}\right)}{w}$$

Thus, all parasitic parameters of the device under test (DUT) are obtained through calculation formulas. The obtained values are used as initial values, and an optimization algorithm is applied for iteration. The final results are shown in Table 2 below.

### 2.3. Extraction of Intrinsic Parameters

The influence of parasitic resistance and parasitic inductance can be neglected under low-frequency conditions. However, when the operating frequency increases, especially when it enters the millimeter-wave band, the interconnect parasitic inductance on the device can no longer be ignored. Therefore, under high-frequency conditions, we need to extract the intrinsic components by de-embedding the influence of parasitic components; the specific de-embedding process is as follows:

1. Perform on-wafer testing on the device under test (DUT) to obtain the corresponding Y-parameter matrix $Y^{DUT}$.

2. Strip off the parasitic capacitance and substrate resistance to obtain the Y-parameter matrix $Y^{HEMT}$ of the device.

(15) $$Y^{HEMT}=Y^{DUT}-Y^{open}$$

3. Convert the Y-parameter matrix $Y^{HEMT}$ into the Z-parameter matrix $Z^{HEMT}$ and then remove the external parasitic inductance and parasitic resistance to obtain the Z-parameter matrix $Z^{int}$ containing only intrinsic components.

(16) $$Z^{int}=Z^{HEMT}-Z^{short}$$

4. Intrinsic Parameter Extraction Formulas for the Device:

(17) $$C_{gd}=-\frac{Im\left(Y_{12}^{int}\right)}{w}$$

(18) $$C_{gs}=\frac{Im\left(Y_{11}^{int}-Y_{12}^{int}\right)}{w}$$

(19) $$g_{ds}=\frac{1}{Re\left(Y_{22}^{int}\right)}$$

(20) $$g_{m}=\left|Im\left(Y_{21}^{int}\right)-Im\left(Y_{12}^{int}\right)\right|$$

(21) $$\tau =-\frac{1}{w}\tan^{-1}\left[\frac{Im\left(Y_{11}^{int}+Y_{12}^{int}\right)}{Re\left(Y_{11}^{int}+Y_{12}^{int}\right)}\right]$$

## 3. Test Structure and Data Validation

**Figure 4:**
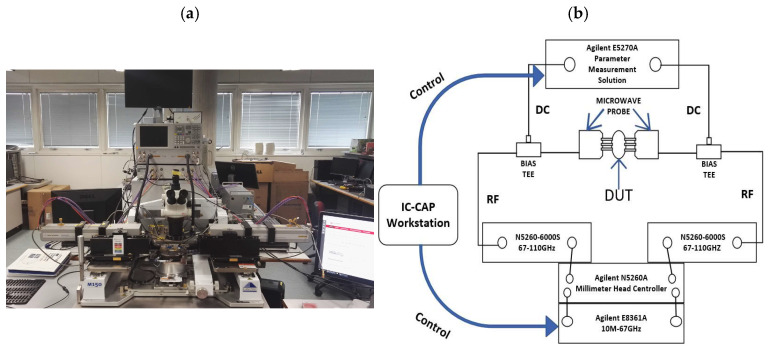
Test System and Test Block Diagram; (**a**) Test System; (**b**) Schematic Diagram of the Test Device.

**Figure 5:**
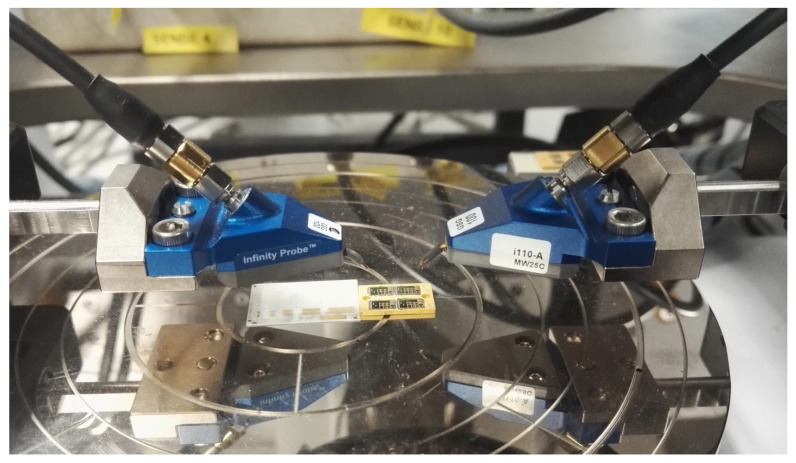
Microwave Probe.

The S-parameter on-wafer measurement system for modeling and verification from 0.5 GHz to 110 GHz was composed of an Agilent E8361A network analyzer (Keysight Technologies, Santa Rosa, CA, USA), an Agilent E5270A parameter measurement solution (Keysight Technologies), a Cacade Probe Station Microtech M150 (FormFactor, Livermore, CA, USA), two Infinity probes GSG100 illo-A MW25c and BiasTee N5260-60013. All measurements were carried out under IC-CAP 2020 software control. The schematic diagram of the on-wafer probe test system and test device is shown in Figure 4, and the microwave probe is shown in Figure 5.

**Figure 6:**
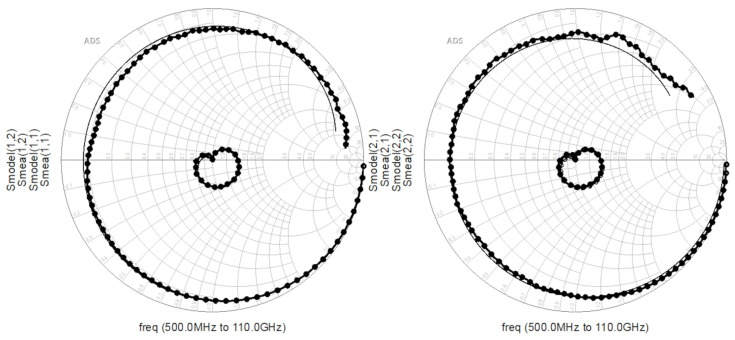
Smith Chart of Measured and Simulated Values for the 2 × 10 Device ( $V_{gs}$ = −5 V, $V_{ds}$ = 8 V).

**Figure 7:**
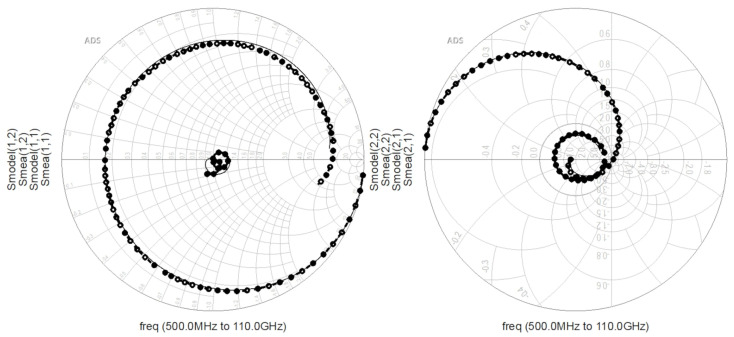
Smith Chart of Measured and Simulated Values for the 4 × 20 Device ( $V_{gs}$ = 0 V, $V_{ds}$ = 16 V).

The intrinsic parameters of two types of devices (2 × 10 and 4 × 20) have been extracted”. The measurement range of $V_{ds}$ is from 0 V to 20 V, and the measurement range of $V_{gs}$ is from −5 V to 0 V. Subsequently, the equivalent circuit illustrated in Figure 2 was constructed in the Keysight ADS 2023 software. The extracted parasitic parameters and intrinsic parameters were substituted into the software as initial values for iterative optimization. The simulation and measurement results of reflection coefficients and transmission coefficients are compared, with the cutoff region of the 2 × 10 device (Figure 6) and the saturation region of the 4 × 20 device (Figure 7) selected, respectively. In the figure, the lines marked with circles are measured values, and the straight lines correspond to simulated values. By comparing the simulated and measured S-parameters, the accuracy of the extracted parameters was verified.

**Figure 8:**
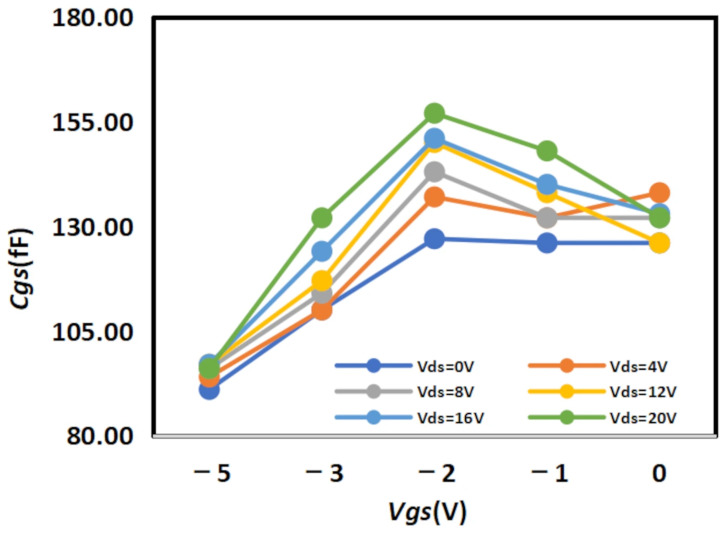
Extracted Results of $C_{gs}$ for 2 × 10 μm.

**Figure 9:**
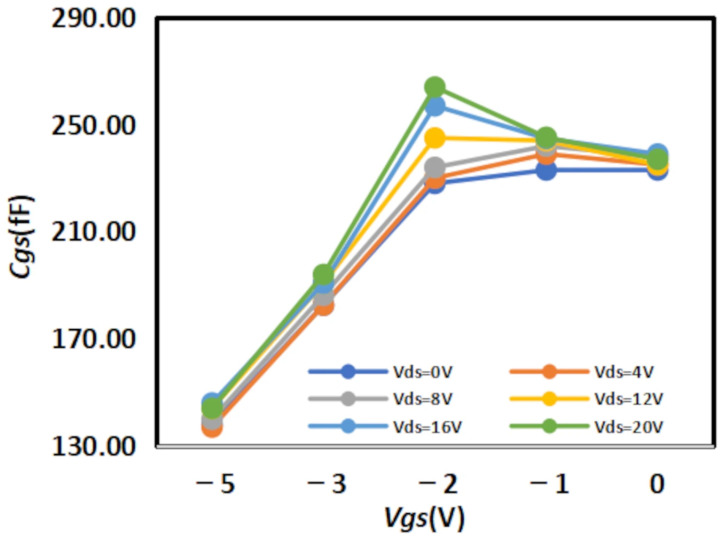
Extracted Results of $C_{gs}$ for 4 × 20 μm.

**Figure 10:**
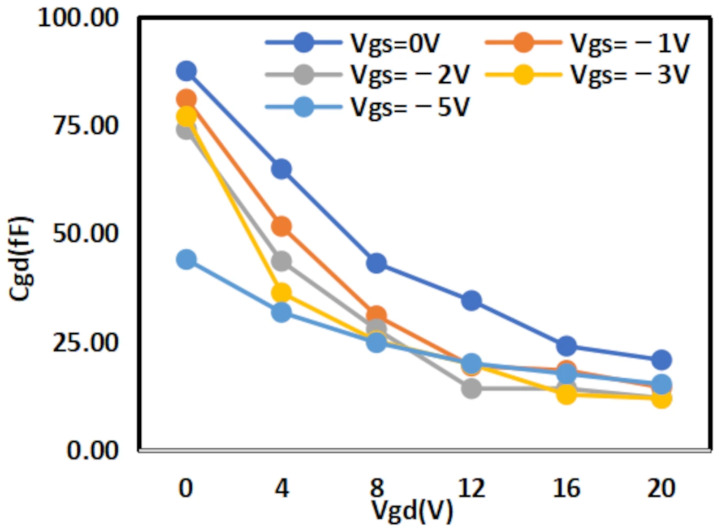
Extracted Results of $C_{gd}$ for 2 × 10 μm.

**Figure 11:**
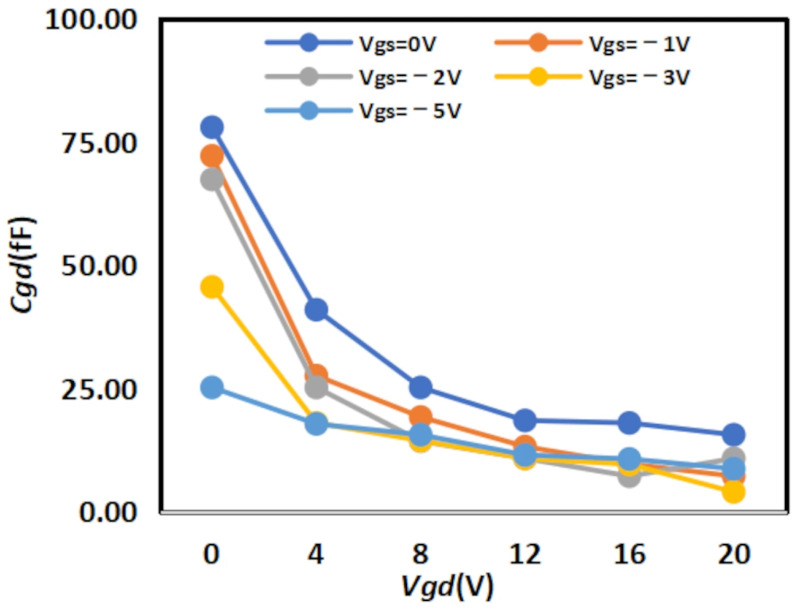
Extracted Results of $C_{gd}$ for 4 × 20 μm.

The extracted $C_{gs}$ results are shown in Figure 8 and Figure 9 below, while the extracted $C_{gd}$ results are shown in Figure 10 and Figure 11 below:

## 4. An Improved Nonlinear C-V Model

### 4.1. Introduction to the Traditional Angelov Model

The Angelov model is a widely used empirical model proposed by Iltcho Angelov in 1992 [13]. Developed based on the Statz and Curtice models, it underwent two major improvements by him in 1996 [15] and 1999 [16], respectively. The effectiveness of this model has been verified in numerous papers and practical applications; it can be well applied to high-frequency applications, blocking effect modeling, and electrothermal effect modeling. It also shows good adaptability in the field of machine learning.

The Angelov model adopts nonlinear $C_{gs}$ and $C_{gd}$ capacitance models, whose expressions are shown as follows:

(22) $$C_{gs}=C_{gso}\left[1+\tanh{(\varphi }_{1})\right]\left[1+\tanh{(\varphi }_{2})\right]$$

(23) $$C_{gd}=C_{gdo}\left[1+\tanh{(\varphi }_{3})\right]\left[1-\tanh{(\varphi }_{4})\right]$$

And

(24) $$\varphi_{1}=P_{10}+P_{11}V_{gs}+P_{12}V_{gs}^{2}+P_{13}V_{gs}^{3}+\cdots$$

(25) $$\varphi_{2}=P_{20}+P_{21}V_{ds}+P_{22}V_{ds}^{2}+P_{23}V_{ds}^{3}+\cdots$$

(26) $$\varphi_{3}=P_{30}+P_{31}V_{gs}+P_{32}V_{gs}^{2}+P_{33}V_{gs}^{3}+\cdots$$

(27) $$\varphi_{4}=P_{40}+P_{41}V_{ds}+P_{42}V_{ds}^{2}+P_{43}V_{ds}^{3}+\cdots$$

Parameters such as $P_{10},P_{11},P_{12},P_{13}$ are all fitting factors.

When the capacitance accuracy can be ensured within the range of 5% to 10%, a first-order approximation can be used to characterize the capacitance:

(28) $$C_{gs}=C_{gsp}+C_{gso}\left[1+\tanh\left(P_{10}+P_{11}V_{gs}\right)\right]\left[1+\tanh\left(P_{20}+P_{21}V_{ds}\right)\right]$$

(29) $$C_{gd}=C_{gdp}+C_{gdo}\left[1+\tanh\left(P_{30}+P_{31}V_{ds}\right)\right]\left[1+\tanh\left(P_{40}+P_{41}V_{gd}\right)\right]$$

### 4.2. Improved Angelov Capacitance Model

Based on the derivation process of the Angelov empirical model, we assume that the gate-source capacitance mainly consists of two components. To simplify the process, we assume that the $C_{gs}$ gate-source capacitance is a function of both $V_{gs}$ and $V_{ds}$ [17], and the following expression can be obtained:

(30) $$C_{gs}\left(V_{gs},V_{ds}\right)=C_{gsp}+C_{gs0}\times f\left(V_{gs}\right)\times g\left(V_{ds}\right)$$

By analyzing the extracted curve of $C_{gs}$ versus $V_{gs}$, it can be observed that $C_{gs}$ does not increase monotonically with $V_{gs}$. Instead, after increasing to a certain extent, $C_{gs}$ begins to decrease. This phenomenon is primarily attributed to the intrinsic nature of GaN HEMTs: as AlGaN/GaN heterojunction transistors, their core physical foundation lies in the spontaneous formation of a two-dimensional electron gas (2DEG) layer with high concentration and high mobility at the AlGaN-GaN interface [18]. This 2DEG layer is not only a key characteristic distinguishing GaN HEMTs from traditional Si MOSFETs and GaAs HEMTs but also the root cause of their “high electron mobility” property. The magnitude of $C_{gs}$ is determined by the concentration and spatial distribution of 2DEG, while the state of 2DEG exhibits a quantum confinement process of “filling-saturation-overflow” as $V_{gs}$ changes [19]. In contrast, the hyperbolic tangent function (tanh) used in traditional models possesses a mathematical property of “monotonic increase/decrease,” which can only describe the “monotonic saturation process from low capacitance to high capacitance” but fails to capture the $C_{gs}$ decrease caused by 2DEG overflow. Notably, this limitation is not a result of insufficient mathematical precision, but rather a mismatch between the function form and the underlying physical mechanism. In comparison, the “S-shaped curve” of the exponential function inherently exhibits non-monotonic characteristics of “increase-plateau-decrease,” which directly corresponds to the quantum behavior of 2DEG. Therefore, the exponential function is selected to replace the hyperbolic tangent function in the original formula.

(31) $$f\left(V_{gs}\right)=\frac{1}{1+e^{-P_{10}\times V_{gs}+P_{11}}}$$

$P_{10},\;P_{11}$ are fitting parameters.

For the second term regarding the influence of $V_{ds}$ on $C_{gs}$, we made modifications based on the original Angelov model by adding the dependence of $C_{gs}$ on $V_{gs}$.

(32) $$g\left(V_{ds}\right)=1+\tanh\left(P_{20}+P_{21}V_{gs}+P_{22}V_{ds}\right)$$

$P_{20},\;P_{21},P_{22}$ are fitting parameters.

In the formula of the original Angelov model, the two-voltage effect is merely a simple product superposition, which only considers the influences of and independently. However, in the actual operation of GaN HEMTs, $V_{gs}$ and $V_{ds}$ mutually modulate each other through the “channel electric field” [20]. Thus, the independent superposition approach adopted in traditional models fails to reproduce the “capacitance coupling effect induced by channel electric field modulation”. Therefore, on the basis of the improved model, we further introduce a cross-coupling term related to $V_{gs}$ and $V_{ds}$. It specifically reproduces the synergistic modulation effect induced by $V_{gs}$ and $V_{ds}$ through the channel electric field and polarization charge distribution [21]. After introducing the cross-coupling term, as $V_{gs}$ increases, the value of the product term rises and the hyperbolic tangent (tanh) function value approaches 1, offsetting the inhibitory effect of $V_{ds}$ on $C_{gs}$. When $V_{gs}$ decreases, the product term value reduces and the tanh function value approaches 0, enhancing the inhibitory effect of $V_{ds}$ on $C_{gs}$. This is fully consistent with the physical reality that “gate control capability dominates channel electric field modulation”.

The improved capacitance model for $C_{gs}$ is as follows:

(33) $$C_{gs}=C_{gsp}+C_{gso}\times \frac{1}{1+e^{-P_{10}\times V_{gs}+P_{11}}}\;\times \left(1+\tanh\left(P_{20}+P_{21}V_{gs}+P_{22}V_{ds}\right)\right)\;\times \tanh\left(P_{23}+P_{24}V_{gs}V_{ds}\right)$$

$P_{10},P_{11},P_{20},P_{21},P_{22},P_{23},P_{24}$ are fitting parameters.

**Figure 12:**
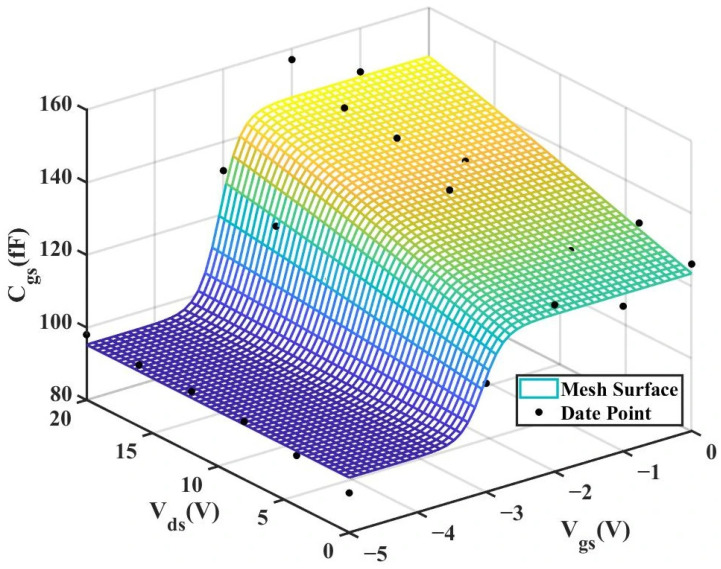
3D $C_{gs}$ (Original 2 × 10 μm Model).

**Figure 13:**
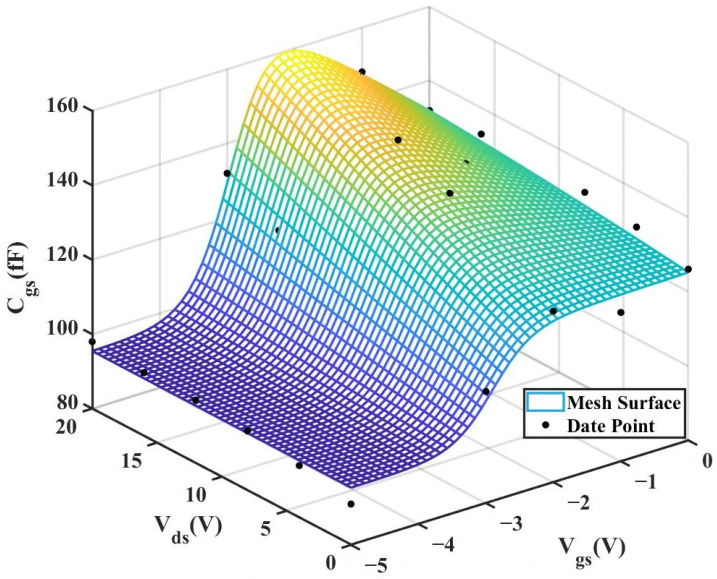
3D $C_{gs}$ (Improved 2 × 10 μm Model).

**Figure 14:**
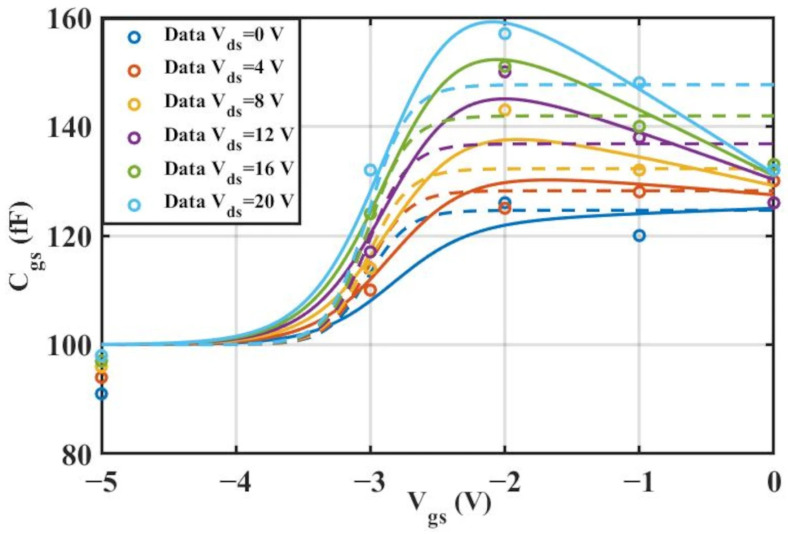
Comparison of Original and Improved Models for $C_{gs}$ vs. $V_{gs}$ (2 × 10 μm).

Substituting the measured data of 2 × 10 μm, the fitting results are shown in Table 3 below:

The experimental relative errors and root mean square errors are shown in Table 4 below:

According to Table 4 above, we can observe that after introducing the cross-coupling term, the improved model achieves a better fitting effect with the original data, and both R^2^ (relative error) and RMSE (root mean square error) show significant improvements. Figure 12 and Figure 13 below present the 3D plot of $C_{gs}$ for improved model and the comparison chart of cross-sections of $C_{gs}$ versus $V_{gs}$ for the original model and the improved model. Figure 14 shows a comparison of the $C_{gs}$ cross-sections between the two models (The solid and dashed lines of different colors in the figure represent different values of $V_{ds}$).

To ensure charge conservation, the formula for the $C_{gd}$ capacitance model is as follows:

(34) $$C_{gd}=C_{gdp}+C_{gdo}\times \frac{1}{1+e^{-P_{30}\times V_{gd}+P_{31}}}\;\times \left(1+\tanh\left(P_{40}+P_{41}V_{gd}+P_{42}V_{ds}\right)\right)\;\times \tanh\left(P_{43}+P_{44}V_{gd}V_{ds}\right)$$

$P_{30},P_{31},P_{40},P_{41},P_{42},P_{43},P_{44}$ are fitting parameters.

**Figure 15:**
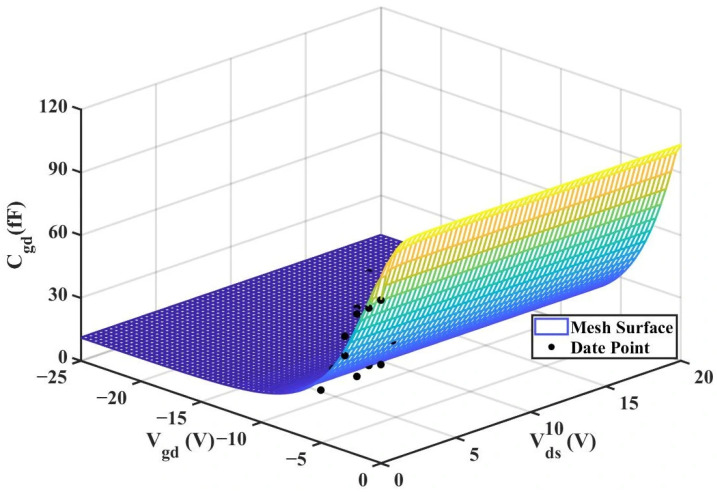
3D $C_{gd}$ (Improved 2 × 10 μm Model).

By substituting the experimental data of 2 × 10 μm, the fitting results are shown in Table 5 and the RMSE and R^2^ values of the two models are shown in Table 6 below:

Experimental comparisons show that the new model has a significantly better fitting effect on the $C_{gd}$ capacitor than the traditional model. The 3D surface plot of the improved $C_{gd}$ model is shown in Figure 15.

It can be observed from the above experiments that the new model has a significantly better fitting effect on the intrinsic capacitor than the traditional model, and its advantage is even more prominent when fitting the nonlinear characteristics of the intrinsic capacitor $C_{gs}$. It is much closer to the measured data values and achieves a far better fitting effect. Both R^2^ (Coefficient of Determination) and RMSE (Root Mean Square Error) have shown significant improvements.

### 4.3. Further Validation of the Model

To more intuitively demonstrate the accuracy of the improved empirical model, we selected the experimental data of 4 × 20 μm to further verify the feasibility and accuracy of the mode. The fitting results of the parameters and the experimental comparison results are shown in Table 7 and Table 8 below.

**Figure 16:**
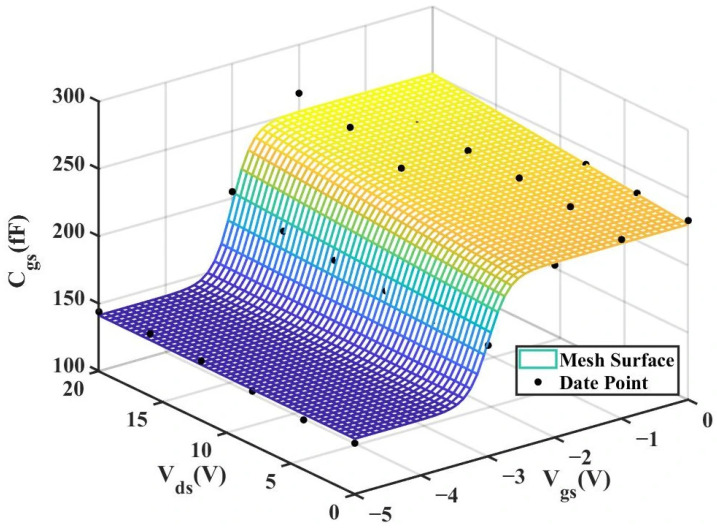
3D $C_{gs}$ (Original 4 × 20 μm Model).

**Figure 17:**
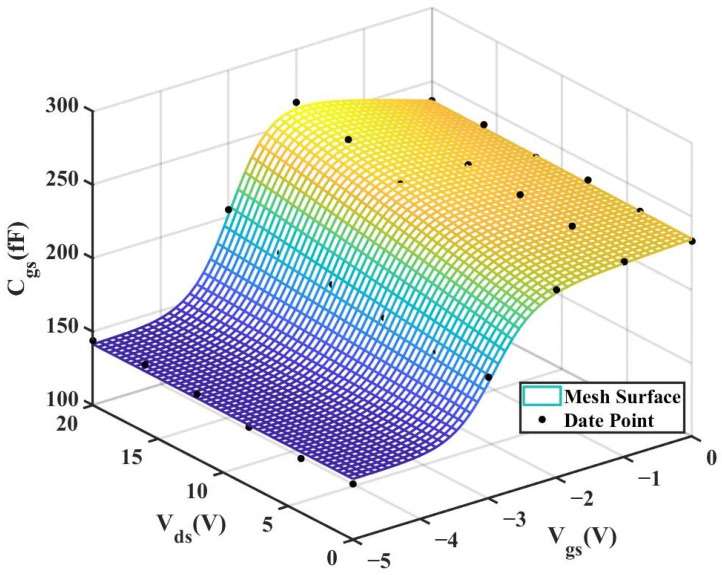
3D $C_{gs}$ (Improved 4 × 20 μm Model).

**Figure 18:**
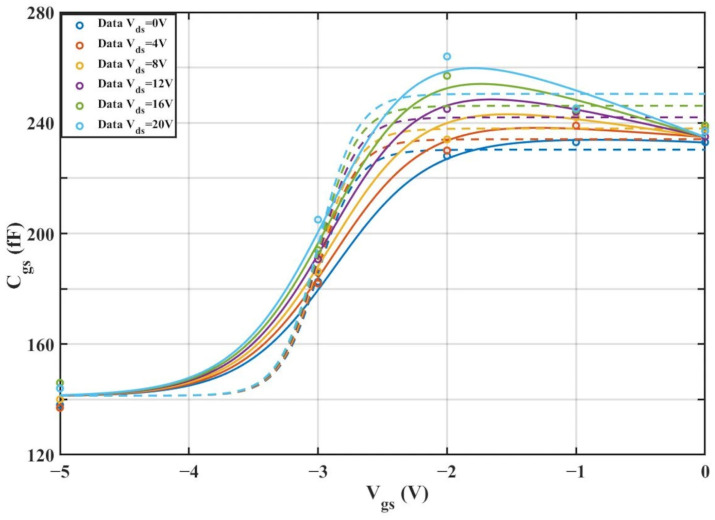
Comparison of Original and Improved Models for $C_{gs}$ vs. $V_{gs}$ (4 × 20 μm).

To validate the improved model, the 3D capacitance surfaces of the 4 × 20 μm device for both the original and improved Angelov models are illustrated in Figure 16 and Figure 17, respectively. A comparative analysis of their 2D cross-sections ( $C_{gs}$ vs. $V_{gs}$) is further provided in Figure 18.

From the above experiments, it can be observed that the improved model still exhibits excellent performance under the dimension of 4 × 20 μm. Compared with the traditional Angelov model, the improved model features higher precision and a better fitting effect with the actual values, enabling it to more accurately depict the nonlinear capacitance characteristics of GaN HEMTs.

We then input the experimental data of $C_{gd}$ (with a size of 4 × 20 μm) into the new model. The optimized parameter values are shown in Table 9 below, and the experimental comparison results are presented in Table 10 below.

**Figure 19:**
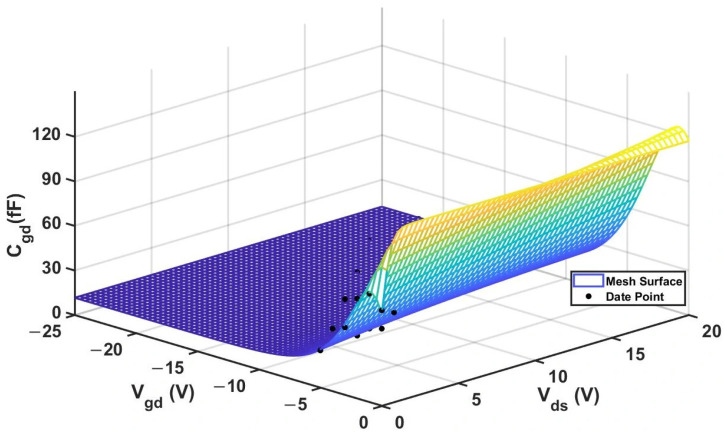
3D $C_{gd}$ (Improved 4 × 20 μm Model).

By comparing the results of the $C_{gd}$ comparison, we can also find that the improved model exhibits a much better fitting effect than the traditional Angelov model, and the fitting results are closer to the actual values. Figure 19 below shows the 3D surface plot of $C_{gd}$.

## 5. Conclusions

This paper mainly focuses on the parameter extraction of GaN HEMTs, with a key emphasis on presenting an improved nonlinear capacitance model based on the Angelov model. Drawing on the derivation method of empirical models, the proposed improved model introduces exponential functions and cross-coupling terms of $C_{gs}$ and $C_{gd}$ to optimize the traditional Angelov model.

Experiments were conducted using two devices of different sizes to compare the performance of the two models, with evaluation metrics including R^2^ (relative error) and RMSE (root mean square error). The experimental results demonstrate that the improved model can more accurately describe the nonlinear characteristics of the intrinsic capacitance. This work contributes to enhancing the accuracy and efficiency of GaN HEMT signal modeling, offering both theoretical significance and practical value.

## Figures and Tables

**Table 1:** Comparison of Various Models.

Model Name	Core Features& Advantage	Disadvantages
**Deep Cutoff Quasi-Physical Capacitance Model** [5]	Covering the entire operating region of the transistor and significantly enhancing the continuity and practicality of modeling.	Equation derivation is complex, with heavy computational load and debugging requiring solid device physics knowledge.
**Simplified Angelov Model (High** ***V_ds_*****)** [6]	In high $V_{ds}$ scenarios, relating $C_{gd}$ and $C_{gs}$ only to $V_{gs}$ significantly reduces the fitting workload and technical threshold for model development.	Narrow applicable scenarios (only for high $V_{ds}$).
**EEHEMT Improved Scalable Model** [7]	Low modeling difficulty and proposes intrinsic capacitance coefficient scaling rules, making it well-suited for large gate-width devices.	Capacitance parameters lack physical meanings, poor scaling rule universality, narrow application scope.
**Dynamic Gate Capacitance Empirical Model** [8]	Having a clear physical mechanism, accounting for dynamic dependence on both $V_{ds}$ and $V_{gs}$ and incorporating physical processes like 2DEG accumulation.	Large number of parameters, complex equations, high computational effort.

**Table 2:** Parasitic Parameter Values.

Parameter Name	Unit	Value
$C_{pgd}$	fF	0.0035
$C_{pg}$	fF	18.4245
$C_{pd}$	fF	16.1755
$R_{pg}$	Ω	43.54
$R_{pd}$	Ω	21.83
$R_{d}$	Ω	5.1713
$R_{g}$	Ω	2.3050
$R_{s}$	Ω	0.315
$L_{d}$	pH	116.232
$L_{g}$	pH	117.655
$L_{s}$	pH	45.334

**Table 3:** Fitting Results of $C_{gs}$ Parameters for 2 × 10 μm.

	$C_{gsp}$	$C_{gso}$	$P_{10}$	$P_{12}$	$P_{20}$	$P_{21}$	$P_{22}$	$P_{23}$	$P_{24}$
Original Model	94.6069	16.8038	10.0000	3.2842	−0.1135	0.0351			
Improved Model	95.4065	162.4434	3.4448	−10.0000	0.4466	0.0164	0.0257	0.1297	−0.0028

**Table 4:** Experimental Result Comparison of $C_{gs}$ for 2 × 10 μm.

	RMSE	R^2^
Original Model	6.0356	0.8910
Improved Model	2.8569	0.9749

**Table 5:** Fitting Results of $C_{gd}$ Parameters for 2 × 10 μm.

	$C_{gdp}$	$C_{gdo}$	$P_{30}$	$P_{31}$	$P_{40}$	$P_{41}$	$P_{42}$	$P_{43}$	$P_{44}$
Original Model	85.1504	−18.4612	0.0274	0.1613	−0.0676	0.2256			
Improved Model	11.0906	200.0000	0.3607	1.1183	0.4799	−0.6994	−1.0741	1.6941	−1.2114

**Table 6:** Experimental Result Comparison of $C_{gd}$ for 2 × 10 μm.

	RMSE	R^2^
Original Model	7.0274	0.8468
Improved Model	3.5002	0.9663

**Table 7:** Fitting Results of $C_{gs}$ Parameters for 4 × 20 μm.

	$C_{gsp}$	$C_{gso}$	$P_{10}$	$P_{12}$	$P_{20}$	$P_{21}$	$P_{22}$	$P_{23}$	$P_{24}$
Original Model	141.3557	84.5615	10.0000	3.3358	−0.5159	0.0072			
Improved Model	141.4108	206.5773	3.4057	−10.0000	1.9190	0.1317	0.0094	0.2507	−0.0023

**Table 8:** Experimental Result Comparison of $C_{gs}$ for 4 × 20 μm.

	RMSE	R^2^
Original Model	5.5671	0.9809
Improved Model	3.1503	0.9946

**Table 9:** Fitting Results of $C_{gd}$ Parameters for 4 × 20 μm.

	$C_{gdp}$	$C_{gdo}$	$P_{30}$	$P_{31}$	$P_{40}$	$P_{41}$	$P_{42}$	$P_{43}$	$P_{44}$
Original Model	115.1728	−25.2927	0.3977	0.0793	0.3394	0.1868			
Improved Model	2.8320	127.9327	0.1124	0.7004	9.5416	−2.4852	1.6179	5.0000	−1.4273

**Table 10:** Experimental Result Comparison of $C_{gd}$ for 4 × 20 μm.

	RMSE	R^2^
Original Model	5.7687	0.9317
Improved Model	4.3875	0.9624

## Data Availability

The simulation and test data used to support the findings of this study are included within the figure files and can be obtained by contacting the author (chengjl@jou.edu.cn).
